# Response to: Education of doctors providing service to LGBTQ patients

**Published:** 2015-12-11

**Authors:** Brenda L. Beagan, Erin Fredericks, Mary Bryson

**Affiliations:** 1 School of Occupational Therapy, Dalhousie University, Halifax, NS; 2 Sociology Department, St. Thomas University, Fredericton, NB; 3 Institute for Gender, Race, Sexuality, and Social Justice, University of British Columbia, Vancouver, BC

We want to thank this group of scholars and practitioners^1^ for their comments on our recent paper,^2^ and thank the editor for giving us the opportunity to respond to those comments. We completely agree with the points being made in the letter.

We agree that gathering perspectives only from health professionals is inherently limited. The paper we published in *CMEJ* 6(1) drew on data from a larger qualitative study, which – as we noted in the paper – included interviews with nurses and with LGBTQ patients. Most good qualitative research produces far more data than can be analyzed in a single paper. Accordingly, we have published several other papers from the study. Some of those focus on the experiences of nurses and physicians,^3^,^4^ some on the experiences of the LGBTQ participants,^5^–^8^ and some on both.^9^ Some specifically teased out the experiences of and with transgender participants for group specific analyses.

We also completely agree that physicians (and other health professionals) need training regarding working effectively with LGBTQ populations, not only in entry-to-practice programs, but also through continuing education for practitioners.

We want to emphasize that at either level training in communication skills and in patient-centered care – while invaluable – is not sufficient. Skillfully treating each patient as an individual may still ignore social group differences that continue to have predictable (though not inevitable) effects on health, health care and everyday experiences. Developing the skills to recognize and explore important social differences, as well as to recognize the biases, assumptions and limitations connected to one’s own social and cultural background are essential first steps toward more equitable health care.
